# How to manage tension gastrothorax: a case report of tension gastrothorax with multiple trauma due to traumatic diaphragmatic rupture

**DOI:** 10.1186/s12245-017-0131-1

**Published:** 2017-01-26

**Authors:** Naofumi Bunya, Keigo Sawamoto, Shuji Uemura, Takashi Toyohara, Yukino Mori, Ryoko Kyan, Kei Miyata, Hideto Irifune, Keisuke Harada, Eichi Narimatsu

**Affiliations:** Department Emergency Medicine, Sapporo Medical University, S1W16 Chuo-ku, Sapporo, Hokkaido 060-8543 Japan

**Keywords:** Tension gastrothorax, Obstructive shock, Resuscitative thoracotomy, REBOA

## Abstract

**Background:**

Tension gastrothorax is a kind of obstructive shock with prolapse and distention of the stomach into the thoracic cavity. Progressive gastric distension leads to mediastinal shift, reduced venous return, decreased cardiac output, and ultimately cardiac arrest. Therefore, it is crucial to decompress the stomach distension for the initial resuscitation of tension gastrothorax.

**Case presentation:**

A 75-year-old female was transported to our resuscitation bay due to motor vehicle crash. At the time of arrival to our hospital, the patient developed cardiac arrest. While undergoing cardiopulmonary resuscitation, an unstable pelvic ring was recognized, so we performed a resuscitative thoracotomy to control hemorrhage and to perform direct cardiac massage. Once we performed the thoracotomy, the stomach and omentum prolapsed out of the thoracotomy site and through the diaphragm rupture site and spontaneous circulation was recovered. Neither the descending aorta nor the heart was collapsed. Although we had continued the treatment for severe pelvic fracture (including blood transufusions), the patient died. Given that (1) the stomach prolapsed out of the body at the time of the thoracotomy; (2) at the same timing, spontaneous circulation returned; and (3) the descending aorta and heart did not collapse, we hypothesized that the main cause of the initial cardiac arrest was tension gastrothorax.

**Conclusions:**

Recognition of tension gastrothorax pathophysiology, which is a form of obstructive shock, makes it possible to manage this injury correctly.

## Background

The first description of tension gastrothorax was reported by Ordog et al. in 1984 [1]. They described that a distended stomach in the thoracic cavity through the site of a diaphragm rupture can lead to mediastinum shift. Gastrothorax develops when increased intraabdominal pressure forces the stomach through an acquired or congenital defect in the diaphragm [2]. Accumulation of gastric contents such as air, fluid and foods in the thoracic cavity raise intrathoracic pressure because the abnormally positioned and angulated gastroesophageal junction functions as a kind of one-way valve [2, 3]. This causes progressive mediastinal shift that can lead to respiratory failure, obstructive shock, and cardiac arrest, much like a tension pneumothorax. We report a case of tension gastrothorax which lead to cardiac arrest and introduce our algorithm for the management of tension gastrothorax.

## Case presentation

A 75-year-old female pedestrian was hit by a motor vehicle. Examination by the emergency medical service crew found her heart rate 130/min, systolic blood pressure 84 mmHg, initial oxygen saturation 78% without supplemental oxygen. On the way to our hospital, an emergency physician got into the ambulance, established two intravenous lines and started fluid resuscitation. He noticed that her lung sounds were decreased on both sides.

Upon admission to our resuscitation bay, she had developed cardiac arrest but still breathed spontaneously. While undergoing cardiopulmonary resuscitation, we detected a pelvic fracture on palpation and found no fluid accumulation in the thoracic or abdominal cavities with ultrasound. We then performed a resuscitative thoracotomy in order to clamp the descending aorta and perform direct cardiac massage because we expected that the cause of cardiac arrest was bleeding from the severe pelvic fracture. At the time of the thoracotomy, the stomach and greater omentum prolapsed out of the body and spontaneous circulation was immediately recovered. We also found that the descending aorta and heart were not collapsed, and the heart was beating strongly. Although we were puzzled why the aorta and heart did not collapse despite the expected severe bleeding, we moved on to manage the pelvic fracture.

**Fig. 1:**
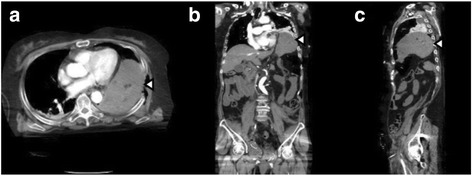
**a**–**c** This computed tomography showed herniation of the stomach through the diaphragm rupture after temporarily closing the thoracotomy incision

While placing a pelvic C clamp to stabilize the pelvic ring and pelvic packing to control bleeding from the retroperitoneal space, we temporarily closed the thoracotomy incision to control wound surface bleeding without clamping the aorta. In spite of these treatments, she gradually became hemodynamically unstable, so resuscitative endovascular balloon occlusion of the aorta (REBOA) was performed. A contrast computed tomography (CT) scan was performed of the head, chest, and abdomen, which revealed multiple trauma, including traumatic subarachnoid hemorrhage, left diaphragm rupture, multiple rib fractures, and a severe pelvic fracture (Fig. 1). After confirming the absence of a basal skull fracture, a nasogastric tube was inserted, but it was unable to reduce gastric contents. To control hemodynamic instability, we performed a transcatheter arterial embolization (TAE) of the internal iliac artery and transformed the patient to the intensive care unit (ICU).

Despite treatment of the severe pelvic fracture with a pelvic C clamp, pelvic packing, and TAE, her hemodynamic instability continued. We considered that the persistent shock was caused by an injury other than pelvic fracture, so we decided to explore the abdominal and thoracic cavities because of the presence of the diaphragmatic injury. As we were unable to maintain adequate hemodynamics in spite of administering massive transfusion protocol and continuous epinephrine infusion, we introduced arterio-venous extracorporeal membrane oxygenation (ECMO). Under ECMO support, an emergency operation was performed with a two-pronged approach with a laparotomy and thoracotomy. Exploring the thoracic and abdominal cavities, we detected only the diaphragm rupture and prolapsed stomach. There was no other obvious intraabdominal organ or thoracic injury. We closed the diaphragm rupture site and chose an open abdominal management to avoid abdominal compartment syndrome. Despite these treatments, the patient died shortly after returning to the ICU. We think that the cause of death was a combination of hemorrhagic shock, traumatic coagulopathy, and post cardiac arrest syndrome caused by the tension gastrothorax.

## Discussion

Tension gastrothorax is considered as a sort of obstructive shock due to the distended stomach expanding into thoracic cavity. Five steps are necessary to develop a tension gastrothorax: (1) existence of a diaphragm defect, (2) increased intraabdominal pressure, (3) prolapse of the stomach into thoracic cavity, (4) a functional change in the gastroesophageal junction (by way of an abnormal angulation) to form a one-way valve, and (5) a reduction in cardiac output as a result of mediastinum shift [2, 3]. These steps might occur simultaneously or the prolapsed stomach might have already existed. This mechanism is similar in tension pneumothorax.

**Fig. 2:**
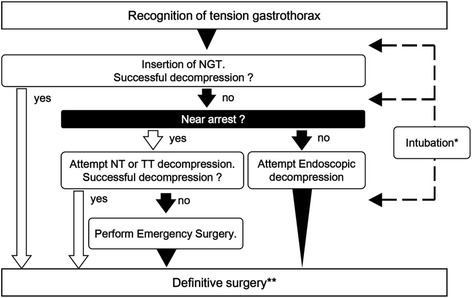
Algorithm of initial resuscitation for tension gastrothorax. *It might be necessary to intubate because of conversion from negative intrathoracic pressure to positive pressure to avoid exacerbation. **After successfully resolving the obstructive shock, definitive surgery can be delayed until the patients respiratory, hemodynamic, and coagulation statuses have stabilized. Definition of abbreviations: *NGT* nasogatric tube, *NT* needle thoracostomy, *TT* tube thoracostomy

The initial resuscitation of tension gastrothorax is to resolve obstructive shock. That is to decompress the distending stomach. A comprehensive literature search was performed using MEDLINE for the year 1984–2015 in order to determine the appropriate initial resuscitation of tension gastrothorax. Search terms were “tension gastrothorax” or “tension viscerothorax”. We excluded literature that did not describe patient hemodynamics or mediastinum shift, and we limited the results to trauma cases. A total of 24 cases were identified, and we reviewed 25 (including our case) to define the initial resuscitation of traumatic tension gastrothorax (Table 1). Four procedures to decompress a traumatic tension gastrothorax were described: (1) insertion of nasogastric tube (NGT), (2) endoscopic approach, (3) needle thoracostomy (NT) or tube thoracostomy (TT), and (4) emergency surgical decompression (thoracotomy or laparotomy). Insertion of a nasogastric tube is recommended in the initial resuscitation of a tension gastrothorax [1], but its placement might be difficult or impossible due to the anatomical change of the gastroesophageal junction [2, 4, 5]. When available, we suggest to perform emergency endoscopic decompression of the gastrothorax. While only described in 3 cases, this method had a 100% success rate in decompression of traumatic tension gastrothorax [4, 6]. If there is insufficient time to perform endoscopic decompression, we suggest to attempt NT or TT [7, 8]. These maneuvers have been tried many times before due to misdiagnoses as tension pneumothorax. However, these methods do not have high success rates and also carry the risk of thoracic cavity contamination. With a tension gastrothorax, however, care must be taken to not penetrate the stomach wall because the distended stomach might be adjacent to the parietal pleura. Finally, if all of these procedures are ineffective, emergency surgery is indicated. Both thoracotomy and laparotomy allow resolution of obstructive shock [5, 9, 10]. The choice of whether to perform a thoracotomy or laparotomy depends on surgeon preference, experience, and individualized patient circumstances. With a laparotomy the surgeon can explore other abdominal organ injuries. With a thoracotomy, it is easier to repair the diaphragm rupture site and allows much quicker resolution of obstructive shock while enabling hemorrhage control deriving from below the diaphragm via clamping of the descending aorta in extreme circumstances. Sidhu et al. reported a case of tension gastrothorax patient saved by urgent thoracotomy [10]. In our case, the resuscitative thoracotomy was effective in restoring spontaneous circulation. Based upon a review of literature, we have proposed an initial resuscitation algorithm for tension gastrothorax (Fig. 2). Following our proposed algorithm, in our case, we should have inserted a nasogastric tube to the depress stomach at the time of the resuscitative thoracotomy.

**Table 1:** Review of tension gastrothorax 25 cases after traumatic injury

		Number (% of all cases)	
Mechanism	Blunt	23 (92%)	
	Penetrate	2 (8%)	
Survive	Yes	19 (76%)	
	No	2 (8%)	
	Unknown	4 (16%)	
Initial	NGT	Success	7 (33%)
Resuscitation		Failure	14 (67%)
	NT	Success	2 (33%)
		Failure	4 (67%)
	TT	Success	1 (20%)
		Failure	4 (80%)
	Endoscopic approach	Success	3 (100%)
		Failure	0 (0%)
	Surgery 9	Laparotomy	6
		Thoracotomy	3

In some cases, initial resuscitation procedures were attempted more than once before resolving tension gastrothorax successfully

Definition of abbreviation: *NGT* nasogatric tube, *NT* needle thoracostomy, *TT* tube thoracostomy

## Conclusions

Recognition of tension gastrothorax pathophysiology, which is a form of obstructive shock, allows emergency and trauma physicians to manage this injury correctly.

## References

[1] Ordog GJ, Wasserberger J, Balasubramaniam S (1984). Tension gastrothorax complicating post-traumatic rupture of the diaphragm. Am J Emerg Med.

[2] Ng J, Rex D, Sudhakaran N, Okoye B, Mukhtar Z (2013). Tension gastrothorax in children: introducing a management algorithm. J Pediatr Surg.

[3] Horst M, Sacher P, Molz G, Willi UV, Meuli M (2005). Tension gastrothorax. J Pediatr Surg.

[4] Abbas A, Thakker M, Booth M, Rechner I (2011). Emergency endoscopic decompression of a delayed posttraumatic tension gastrothorax. Am J Emerg Med.

[5] Ekim H, Tuncer M, Ozbay B (2008). Tension viscerothorax due to traumatic diaphragmatic rupture. Ann Saudi Med.

[6] Bamgbade OA (2006). Management of tension gastrothorax. Resuscitation.

[7] Salim F, Ramesh V (2009). Tension gastrothorax: a rare complication. J Coll Physicians Surg Pak.

[8] Sivrikoz MC, Doner E, Tulay MC (2007). Tension viscerothorax mimicking tension pneumothorax. Am J Emerg Med.

[9] Nishijima D, Zehbtachi S, Austin RB (2007). Acute posttraumatic tension gastrothorax mimicking acute tension pneumothorax. Am J Emerg Med.

[10] Sidhu BS, Mallick SK, Ahluwalia GS (1988). Tension gastrothorax complicating traumatic diaphragmatic hernia. J Indian Med Assoc.

